# Asymptomatic Vivax and Falciparum Parasitaemia with Helminth Co-Infection: Major Risk Factors for Anaemia in Early Life

**DOI:** 10.1371/journal.pone.0160917

**Published:** 2016-08-09

**Authors:** Faustina Helena Burdam, Mohammad Hakimi, Franciscus Thio, Enny Kenangalem, Ratni Indrawanti, Rintis Noviyanti, Leily Trianty, Jutta Marfurt, Irene Handayuni, Yati Soenarto, Nicholas M. Douglas, Nicholas M. Anstey, Ric N. Price, Jeanne Rini Poespoprodjo

**Affiliations:** 1 Mimika District Health Authority, Timika, Papua, Indonesia; 2 Mimika District Hospital, Timika, Papua, Indonesia; 3 Timika Malaria Research Programme, Papuan Health and Community Development Foundation, Timika, Papua, Indonesia; 4 Department of Child Health, Faculty of Medicine, Universitas Gadjah Mada/Dr. Sardjito Hospital, Yogyakarta, Indonesia; 5 Maternal and Child Health and Reproductive Health, Department of Public Health, Faculty of Medicine, Universitas Gadjah Mada, Yogyakarta, Indonesia; 6 Eijkman Institute for Molecular Biology, Jakarta, Indonesia; 7 Global and Tropical Health Division, Menzies School of Health Research and Charles Darwin University, Darwin, Australia; 8 Centre for Tropical Medicine and Global Health, Nuffield Department of Clinical Medicine, University of Oxford, Oxford, United Kingdom; Université Pierre et Marie Curie, FRANCE

## Abstract

**Background:**

Anaemia in children under five years old is associated with poor health, growth and developmental outcomes. In Papua, Indonesia, where the burden of anaemia in infants is high, we conducted a community survey to assess the association between *Plasmodium* infection, helminth carriage and the risk of anaemia.

**Methods:**

A cross sectional household survey was carried out between April and July 2013 in 16 villages in the District of Mimika using a multistage sampling procedure. A total of 629 children aged 1–59 months from 800 households were included in the study. Demographic, symptom and anthropometry data were recorded using a standardized questionnaire. Blood and stool samples were collected for examination.

**Results:**

Of the 533 children with blood film examination, 8.8% (47) had *P*. *vivax* parasitaemia and 3.9% (21) had *P*. *falciparum;* the majority of children with malaria were asymptomatic (94.4%, 68/72). Soil transmitted helminth (STH) infection was present in 43% (105/269) of children assessed; those with STH were at significantly greater risk of *P*. *vivax* parasitaemia compared to those without STH (OR = 3.7 [95%CI 1.5–9.2], p = 0.004). Anaemia (Hb<10 g/dl) was present in 24.5% (122/497) of children and associated with *P*. *vivax* parasitaemia (OR = 2.9 [95%CI, 1.7–4.9], p = 0.001), *P*. *falciparum* parasitaemia (OR = 4.3 [95%CI, 2.0–9.4], p<0.001), hookworm carriage (OR = 2.6 [95%CI, 1.2–5.8], p = 0.026), *Plasmodium*–helminth coinfection (OR 4.0 [95%CI, 1.4–11.3], p = 0.008) and severe stunting (OR = 1.9 ([95%CI, 1.1–3.3], p = 0.012).

**Conclusions:**

Asymptomatic *P*. *vivax* and *P*. *falciparum* infections and hookworm all contribute to risk of paediatric anaemia in coendemic areas and should be targeted with prevention and treatment programs. The relationship between helminth infections and the increased risk of *P*. *vivax* parasitaemia should be explored prospectively.

## Introduction

Anaemia remains a critical determinant of global morbidity and mortality in children under five years old [1–3]. Whilst mild anaemia is usually asymptomatic and may go unrecognized by both patients and healthcare providers [4], it has been associated with impaired immune function [5–8]. Anaemic individuals in poorly resourced communities are vulnerable to a cycle driven by low host immunity, an increased susceptibility to malnutrition and infection, which give rise to further haemopoietic suppression and haemolysis. As the hemoglobin concentration falls below 8g/dl the risk of mortality rises [1, 9]. In those who do not succumb, the consequences of severe anaemia can be long-lasting, including cognitive impairment, stunting and delays in motor development [10–12].

The aetiology of anaemia in malaria-endemic areas is multifactorial. Risk factors include parasite-induced haemolysis and haemopoietic suppression, iron deficiency, poor nutritional intake, recurrent bacterial infections and soil transmitted helminthiases [13] [2, 9]. Whilst the health consequences of asymptomatic falciparum malaria and its interplay with other causes of anaemia has been relatively well defined in children in *P*. *falciparum* endemic areas of Africa [4, 14–16], the interplay between *P*. *vivax* infection and stool transmitted helminth (STH) infections is poorly understood with little known about the factors associated with anaemia attributable to *P*. *vivax* [17].

Observational studies in Papua province in eastern Indonesia, have demonstrated that severe anaemia (Hb<5 g/dl) is present in almost 20% of young children attending an inpatient healthcare facility; approximately a third of this anaemia attributable to malaria [3, 9]. Papua, is a malaria endemic region unusual in having high prevalence of antimalarial drug resistance in both *P*. *falciparum* and *P*. *vivax* [18, 19]. Recurrent clinical episodes and sustained parasitaemia are key contributing factors and occur because of treatment failure (recrudescence), reinfection and relapsing infections from the dormant liver stages of *P*. *vivax*.

The prevalence of STH in Papua has not been well characterized, nor its role in *P*. *vivax* co-infections and in the aetiology of anaemia. In the current cross sectional survey, the relationship between *Plasmodium* infection, nutritional status, helminth coinfection and anaemia was assessed to provide the evidence base for suitable intervention strategies.

## Methods

### Study site

The district of Mimika in the southern part of Papua Province, Indonesia has both highland and lowland areas. According to the local census, the population was 202,350 in 2012 with almost 90% of the population located in the lowlands, which is largely forested with a climate that varies little throughout the year [20]. There are 12 sub-districts and 79 villages in an area of 19,602 km^2^, with the largest three sub-districts reachable by land from the capital city (Timika).

Malaria in the region is restricted to lowland areas where it is associated with three mosquito vectors: *Anopheles koliensis*, *An*. *farauti* and *An*. *punctulatus*. The area has unstable malaria transmission with an estimated annual incidence of parasitaemia of 450 per 1000 population in 2013, divided 60:40 between *P*. *falciparum* and *P*. *vivax* infections [21]. Clinical trials in 2004 revealed extremely high antimalarial treatment failure, with 65% of patient having recurrent parasitaemia 28 days after chloroquine (CQ) monotherapy for *P*. *vivax* and 48% on day 42 after CQ plus sulfadoxine-pyrimethamine (SP) for *P*. *falciparum* [18]. As previously mentioned, the first line treatment policy for uncomplicated malaria in Mimika was changed in March 2006 to dihydroartemisinin-piperaquine (DHP) for malaria due to any *Plasmodium* species [22]. Insecticide treated nets are used by approximately 52% of the population [21].

Soil transmitted helminthiases (STHs) are prevalent in malaria-endemic areas, but precise data in this region are lacking. There is no specific program for deworming or iron supplementation in young children in the district.

### Study population

Children aged less than 5 years old were enrolled in this study as part of a larger household survey that included all consenting members of the households. This age group accounts for 15% of the local population [20]. The under five year mortality rate in this region is 115/1000 live births with a neonatal mortality rate of 27/1000 live births [23]. Malaria is the main cause of outpatient and inpatient visits to the local hospital in under fives, followed by diarrhea and lower respiratory tract infections [24].

The ethnicity of the population can be broadly categorized into highland and lowland Papuans and non-Papuans.

### Study design

A cross sectional survey was carried out between April and July 2013 in 16 villages in 3 sub-districts in the District of Mimika to evaluate the impact of malaria treatment policy change to malaria prevalence as described previously [19]. Surveys were conducted before and after treatment policy change in March 2006. The first survey was conducted in 2005 and the second one in 2013.

The survey sample size was powered to detect a 40% reduction in malaria prevalence assuming an estimated incidence of malaria of 500 episodes/1000 people/year; a total of 4000 participants were required. In the District of Mimika, each household consists of approximately 5 persons and this amounted to 800 households. Children under-fives in the households were included in the study.

A multistage sampling procedure was applied to identify clusters of 25 households. Firstly the three largest sub-districts were chosen purposively from the 12 sub-districts based on their accessibility and inclusion of the residence of the majority (80%) of the population. The proportions of ethnicity and socioeconomic strata was similar between this three subdistricts [20]. Secondly, the number of clusters in each sub-district was apportioned according to the relative populations of the subdistricts. The houses were then assigned numbers according to their geographical location and clusters of 25 houses were identified randomly according to WHO guidelines with the first house identified by random allocation [25].

A total of 32 clusters of 25 randomly selected households were included in the survey providing a total of 800 households. Household members were defined as people who lived under one roof, ate from one kitchen and who had resided in the study area for at least 6 months (except for infants younger than 6 months).

For each household member, sociodemographic information and history of fever were recorded using a standardised questionnaire. Socioeconomic status was defined according to the national standard of minimal employer’s wage in the region. Those present at the time of the survey had their weight, height and temperature recorded and a finger prick sample of blood taken for blood film examination and haemoglobin measurement. Patients with microscopically confirmed malaria were treated with DHP [26]. Stool samples were collected from all children aged less than 5 years old. The parent/guardian was given a sealed plastic container with stick to collect small amount of stool sample on the first visit for collection on the next day in the morning.

Haemoglobin concentration was determined by electronic coulter counter (Coulter JT^™^, USA). Malaria was defined as the presence of peripheral asexual parasitaemia by microscopy irrespective of clinical signs or *Plasmodium* species. Two trained research microscopists examined the slides independently. Two different microscopists examined the stool samples using the Katz Kato method and recorded both the species present and concentration of eggs per gram of stool according to the guidelines [27, 28]. The final result of parasite and egg counts was the average of the two different counts. If there was a discrepancy in the species diagnosis or a difference in the parasite and egg count greater than 50%, an independent third reader re-examined the slide and final result made according to consensus.

### Definitions

An ‘infant’ was defined as a child aged less than 1 year old and a “young child” if aged 1 to <5 years. Children under-five included both infants and young children. Anaemia was defined as a hemoglobin concentration less than 10 g/dl. Nutritional status was assessed according to the WHO 2007 Standard Reference [29]. Underweight and severe underweight were defined as weight for age Z-scores below– 2SD and– 3 SD respectively. Similar categories were applied to wasting-severe wasting and stunting-severe stunting by examining Z-scores of height/length for weight and height/length for age respectively.

### Statistical analysis

Questionnaire and laboratory data were entered into EpiData 3.02 software (EpiData Association, Odense, Denmark). Data were analysed using SPSS vs 20.0 for windows software (IBM SPSS Statistics). Normally distributed data were compared by Student’s t-test. Data not conforming to a normal distribution were compared by the Mann-Whitney U test. Categorical data were compared by using odds ratios (OR with 95% confidence intervals), the chi squared test with Yates' correction or by Fisher's exact test. Multiple logistic regression was used to analyse independent risk factors for anaemia by entering all significant risk factors with p value <0.05 in univariate analysis.

### Ethical Approval

Ethical approval for this study was obtained from the Medical and Health Research Ethics Committee, Faculty of Medicine, Gadjah Mada University, Yogyakarta, Indonesia (KE/FK/39/EC), Menzies School of Health Research, Darwin, Australia (HREC 10–1434) and Oxford Tropical Research Ethics Committee (44–1). Written consent was obtained from the head of the household, and included consent for all members of the family to participate in the study, including children aged less than 16 years old.

## Results

**Fig 1:**
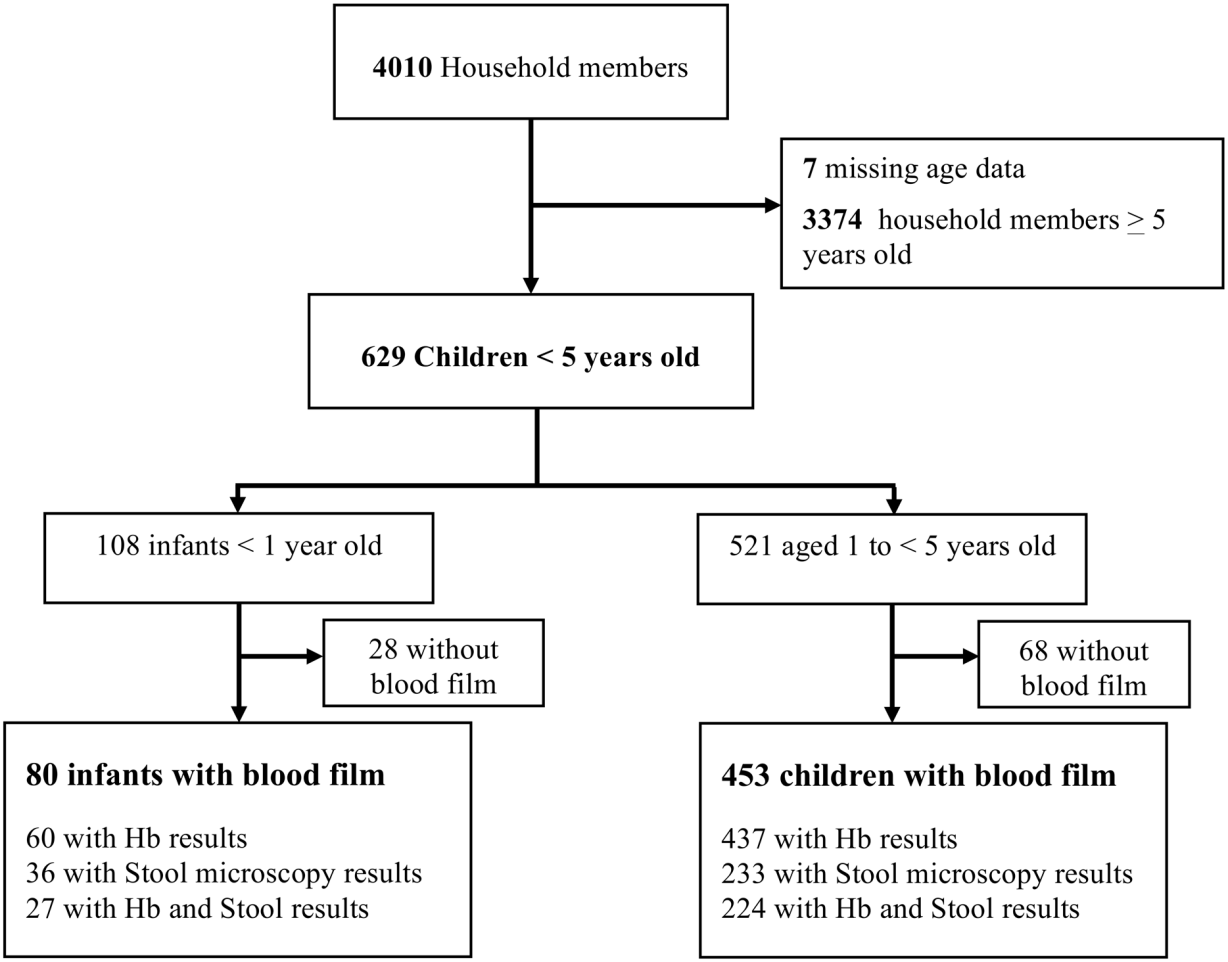
Study Profile.

Between April and July 2013 a total of 629 children aged 1 to 59 months old (median age 24 months, range 1–59 months) were enrolled from 800 households; see Fig 1. Infants accounted for 17% (108) of children under-five. Characteristics of the study participants are presented in Table 1. Of the 531 children with available weight data and the 507 children with length and height data, 76 (14.3%) were classified as underweight, 47 (9.3%) as wasted, and 16.7% (85/507) stunted; these risks were similar between infants and young children and were not associated with ethnicity or socioeconomic status (Table 1).

**Table 1:** Characteristics of the study participants.

Characteristic	Age group (in years)		
	Infants < 1 years, n (%)	Children 1 to < 5 years, n (%)	P value
	**N = 80**	**N = 453**	
Male	41 (51.3)	220 (48.6)	0.72
**Ethnic Group**			
Highland Papuan	16 (20.0)	94 (20.8)	0.97
Lowland Papuan	22 (27.5)	119 (26.3)	
Non Papuan	42 (52.5)	240 (53.0)	
Fever within 24 hours	1 (1.3)	5 (1.1)	1.00
Fever within last 1 month	4 (5.0)	17 (3.8)	0.54
Sleeping under bednet	32 (40)	170 (37.5)	0.71
**Weight for Age Nutritional Status**	N = 79	N = 452	
Underweight	7 (8.9)	69 (15.3)	0.16
Severe underweight	5 (6.3)	18 (4.0)	0.43
**Height for Weight Nutritional Status**	N = 73	N = 434	
Wasting	7 (9.6)	40 (9.2)	1.00
Severe wasting	3 (4.1)	40 (9.2)	0.17
**Height for Age Nutritional Status**	N = 73	N = 437	
Stunting	11 (15.1)	74 (16.9)	0.77
Severe stunting	19 (26.0)	74 (16.9)	0.12
**Haemoglobin data**	N = 60	N = 437	
Mean Haemoglobin, g/dl (95%CI)	10.99 (10.64–11.34)	11.18 (10.08–11.37)	0.006
Anaemia			
Hb<10 gr/dl	13 (21.7)	109 (24.9)	0.64
Hb<7 gr/dl	0 (0)	16 (3.7)	0.24
Hb<5 gr/dl (severe)	0 (0)	0 (0)	n/a
**Plasmodium infection**	N = 80	N = 453	
Any Plasmodium infection	6 (7.5)	66 (14.6)	0.11
Species of infection			
*P*. *falciparum*	2 (2.5)	19 (4.2)	0.55
*P*. *vivax*	4 (5.0)	43 (9.5)	0.21
*P*. *malariae*	0 (0)	1 (0.2)	n/a
Mixed infection	0 (0)	3 (0.7)	n/a
**Helminth infestation**	N = 36	N = 233	
Any worms	7 (19.4)	95 (40.8)	0.01
*Ascaris Lumbricoides*	2 (5.6)	73 (31.3)	<0.0001
*Trichuris trichiura*	2 (5.6)	54 (23.2)	0.01
Hook worm	4 (11.1)	31 (13.3)	1.00

### Peripheral Parasitaemia by microscopy

Blood film examination was undertaken in 85% (533) of children of whom 8.8% (47) had *P*. *vivax*, 3.9% (21) had *P*. *falciparum* parasitaemia, 0.2% (1) had *P*. *malariae* and 0.6% (3) had both *P*. *falciparum* and *P*. *vivax* parasitaemia. Peripheral parasitaemias were significantly higher in *P*. *falciparum* infections (geometric mean = 3789 parasites/μL [95%CI, 1096–12,088]) compared to *P*. *vivax* (982 parasites/μL [95%CI, 601–1635]; p = 0.048) infections and the proportion with fever was also higher for *P*. *falciparum* (14.3%, 3/21 versus 5.6%, 4/72, respectively; p = 0.1).

Three risk factors for *Plasmodium* infection were identified: Lowland Papuan ethnicity (Adjusted Odds Ratio (AOR) 3.1 [95%CI, 1.6–6.0], p<0.001), fever in the last 24 hours (AOR = 12.8 [95%CI, 1.5–111.7], p = 0.021), and anaemia (AOR = 5.1 [95%CI, 2.9–9.2], p<0.001) (Table 2).

**Table 2:** Risk factors for *Plasmodium* infection (any species) in children aged < 5 years old.

Characteristics	Any Malaria, % (n/valid cases)	Univariate analysis		Multivariate analysis	
		OR (95% CI)	P value	AOR (95% CI)	P value
**Sex**					
Male	12.6 (33/261)	0.9 (0.5–1.4)	0.61	-	-
**Ethnic group**					
Non Papuan	7.4 (21/282)	Reference		Reference	
Highland	14.5 (16/110)	2.1 (1.1–4.2)	<0.0001	1.5 (0.7–3.3)	0.28
Lowland	24.8 (35/141)	4.1 (2.3–7.4)	<0.0001	3.1 (1.6–6.0)	<0.0001
**Socioeconomic status**					
Below minimum wage	3.7 (1/27)	1.1 (0.7–1.8)	0.80	-	-
**Age group**					
1 to < 5 years	14.6 (66/453)	Reference		-	-
< 1 year	7.5 (6/80)	0.5 (0.2–1.1)	0.11	-	-
**Nutritional Status**					
Well nourished	*	Reference		-	-
Underweight	7.9 (6/76)	0.5 (0.2–1.3)	0.15		-
Severe underweight	13.0 (3/23)	0.9 (0.3–3.1)	0.84	-	-
Wasting	12.8 (6/47)	0.9 (0.4–2.2)	0.83	-	-
Severe wasting	7 (3/43)	0.5 (0.1–1.6)	0.21	-	-
Stunting	14.1 (12/85)	1.2 (0.6–2.4)	0.65	-	-
Severe stunting	16.1 (15/93)	1.4 (0.7–2.6)	0.33	-	-
**Fever**					
Fever 24 hours	66.7 (4/6)	13.5 (2.4–75.1)	0.004	12.8 (1.5–111.7)	0.02
History of fever	38.1 (8/21)	4.3 (1.7–10.8)	0.004	1.6 (0.5–5.3)	0.44
**Anaemia**					
Hb<10 gr/dl	34.4 (42/122)	6.7 (3.9–11.6)	<0.0001	5.1 (2.9–9.2)	<0.0001
**Helminthiasis**					
Negative	9.6 (16/167)	Reference			
Helminthiasis	18.6 (19/102)	2.2 (1.1–4.4)^#^	0.04	1.5 (0.8–2.8)	0.25

Note:

^#^Risk for *vivax* malaria: OR 3.75 (95%CI, 1.53–9.2), p = 0.004; Risk for *falciparum* malaria: OR 2.0 (95%CI, 0.4–10.1), p = 0.402;

*the proportion depends on nutritional status calculation method used

### Helminth Carriage

Stool examination was undertaken in 269 (43%) children of whom 39% (105) had soil transmitted helminths (STHs); almost half of these children were infested with multiple STHs (49%, 52/105). *Ascaris lumbricoides* (AL), *Trichuris trichiura* (TT) and hookworm were present in 28%, 21% and 13% of the children respectively.

The prevalence of helminthiasis was 19.4% (7/36) in infants compared to 40.8% (95/233) in children aged 1 to 5 years (Tables 1 and 3). The egg burden did not significantly differ with age, however the number of infections in the infant group was small (Table 4). The median [range] age of children infested with AL was 36.0 [6.9–58.8] months compared to 36.0 [3.0–48.0] months for TT and 36.0 [1.0–57.6] months for hookworm. Females were more likely to have STHs than males (OR 1.9 [95%CI 1.2–3.2], p = 0.019) whereas highland Papuans were more likely to have STHs compared to Non Papuans (OR = 4.2 [95%CI, 2.1–8.3], p<0.0001); Table 3. The risk of STH was significantly lower in the 47 children with wasting (OR = 0.2 [95%CI, 0.1–0.8], p = 0.02), although this was not statistically significant after controlling for ethnicity. The risk of having STHs did not vary with socio economic status.

**Table 3:** Risk factors for any helminthiasis in children aged < 5 years old.

Characteristics	Any Helminthiasis, % (n/valid cases)	Univariate analysis		Multivariate analysis	
		OR (95% CI)	P value	AOR (95% CI)	P value
**Sex**					
Female	44.6 (62/139)	1.9 (1.2–3.2)	0.01	1.9 (1.1–3.3)	0.02
**Ethnic group**					
Non Papuan	26.8 (44/164)	Reference		Reference	
Highland	61.7 (29/47)	4.2 (2.1–8.3)	<0.001	4.2 (2.1–8.5)	<0.001
Lowland	44.4 (32/72)	2.0 (1.1–3.7)	0.02	2.1 (1.1–3.8)	0.02
**Socioeconomic status**					
Below minimum wage	42.9 (6/14)	0.8 (0.5–1.3)	0.38	-	-
**Age group**					
< 1 year	17.5 (7/40)	Reference		Reference	
1 to < 5 years	40.3 (98/243)	2.9 (1.2–6.8)	0.02	3.2 (1.3–7.9)	0.01
**Fever**					
Fever 24 hours	50.0 (1/2)	1.7 (0.1–27.5)	1.00	-	-
Fever 1 month	50.0 (6/12)	1.7 (0.5–5.5)	0.37	-	-
**Nutritional Status**					
Well nourished	*	Reference			
Underweight	25.6 (10/39)	0.5 (0.3–1.1)	0.09	-	-
Severe underweight	20.0 (3/15)	0.6 (0.2–1.9)	0.36	-	-
Wasting	15.0 (3/20)	0.2 (0.1–0.8)	0.02	-	-
Severe wasting	27.3 (6/22)	0.5 (0.2–1.5)	0.25	-	-
Stunting	33.3 (15/45)	0.8 (0.4–1.6)	0.47	-	-
Severe stunting	36.8 (21/57)	0.9 (0.5–1.7)	0.75	-	-
**Anaemia**					
Hb<10 gr/dl	48.4 (30/62)	1.7 (0.9–3.0)	0.10	-	-

Note:

*the proportion depends on nutritional status calculation method used

**Table 4:** Helminthiasis Profile.

	Age group		P values
	Infant (< 1 year old)	Underfive children (1 to < 5 years old)	
	N = 40	N = 243	
*Ascaris lumbricoides*, n (%)	2 (3.5)	75 (32.7)	
Median egg count (range), eggs/gram	120 (24–216)	2640 (24–167,728)	0.08
*Trichuris trichiura*, n (%)	2 (3.5)	57 (24.8)	
Median egg counts (range), eggs/gram	84 (24–144)	168 (24–10,224)	0.20
Hookworm, n (%)	4 (7.1)	31 (13.5)	
Median egg counts (range), eggs/gram	396 (24–1584)	360 (24–3696)	0.82

Overall children with STHs were at significantly greater risk of parasitaemia (OR 2.2 [95%CI 1.1–4.4], p = 0.04) although the trend remained in multivariable analysis it was no longer significant (Table 2). The difference was mostly apparent for *P*. *vivax* parasitaemia with 15.3% (15/98) of children with STHs having parasitaemia recorded compared to 4.6% (8/174) of children without STH (OR = 3.7 [95%CI 1.5–9.2], p = 0.004). The risks of *P*. *vivax* infections remained higher compared to those without STH after stratifying by species of STH: *Ascaris lumbricoides*, *Trichuris trichiura* and hookworm with the OR of 3.7 (95%CI, 1.5–9.3, p = 0.01), 3.8 (95%CI, 1.5–9.9, p = 0.01) and 3.5 (95%CI, 1.5–10.5, p = 0.03) respectively.

### Anaemia

**Fig 2:**
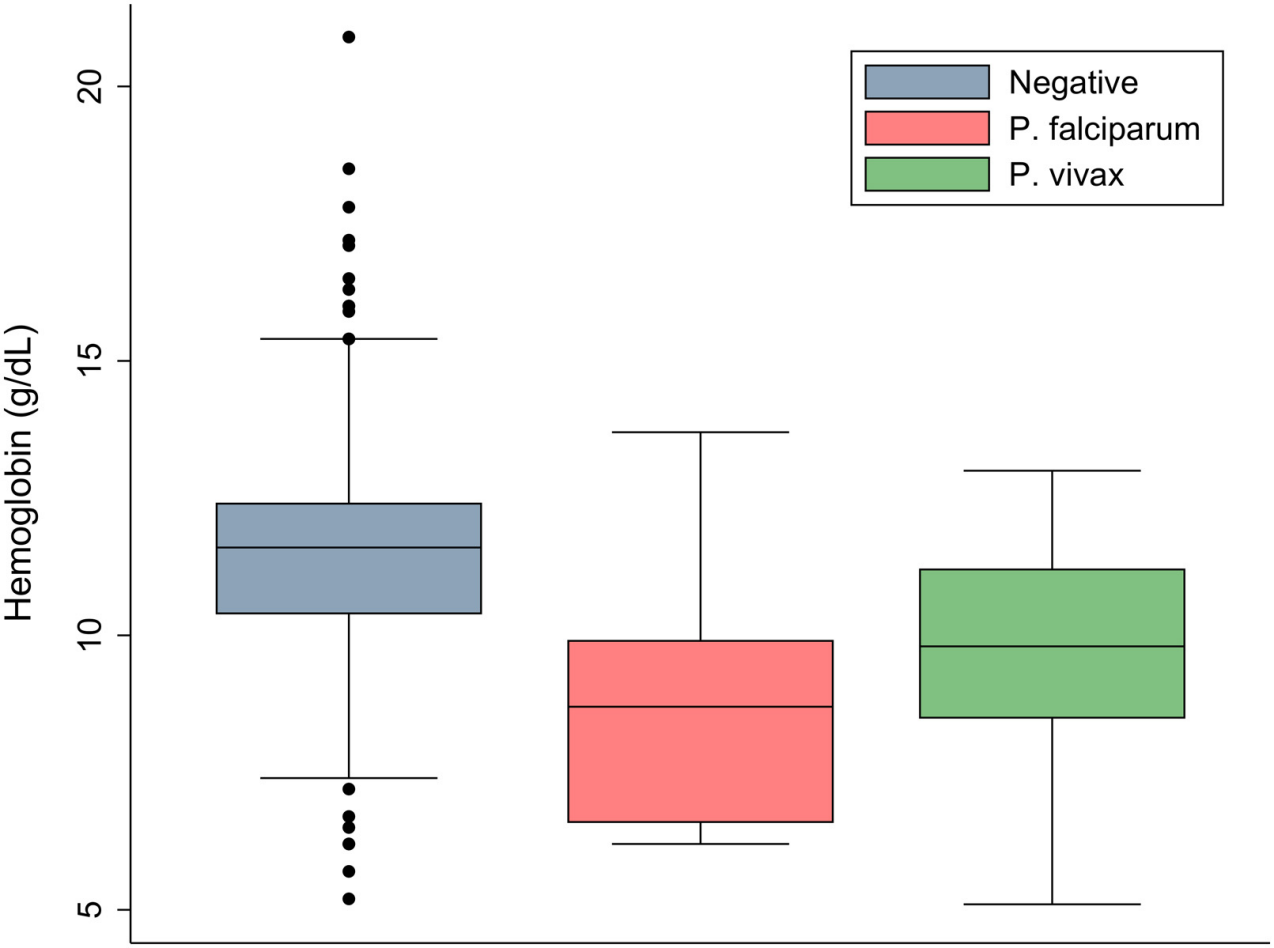
Haemoglobin distribution stratified by parasitaemia status.

Data on haemoglobin concentration were available in 60 (55.5%) infants (mean Hb 10.9 g/dl [95%CI 10.6–11.3]) and 437 (83.8%) young children (mean Hb 11.2 g/dl [95%CI 10.1–11.4]); Fig 2. In total 21.7% (13/60) of infants and 24.9% (109/437) of young children were anaemic (Hb<10g/dl); p = 0.635. Sixteen children (3.2%) had severe anaemia with hemoglobin concentrations between 5.1 and 7 g/dl. Data on mean corpuscular volume (MCV) were available in 51 children with microcytosis (MCV<74 fL) reported in 33 (65%) individuals overall and 75% (9/12) of children with anaemia. Risk factors for anaemia included a history of fever in the last 4 weeks (OR = 3.6 [95%CI, 1.3–10.1], p = 0.015) and Papuan ethnicity (OR = 4.0 [95%CI, 2.5–6.1], p<0.001) compared to non Papuans; Table 5.

**Table 5:** Risk factors for anaemia (Hb< 10 g/dl).

Characteristics	Anaemia, % (n/valid cases)	Univariate analysis		Multivariate analysis	
		OR (95% CI)	P value	AOR (95% CI)	P value
**Sex**					
Male	25.4 (62/244)	1.1 (0.7–1.6)	0.68	-	-
**Ethnic group**					
Non Papuan	13.5 (35/264)	Reference		Reference	
Highland	35.9 (37/103)	3.7 (2.1–6.3)	<0.0001	3.2 (1.8–5.9)	<0.001
Lowland	38.5 (50/130)	4.1 (2.5–6.7)	<0.0001	3.2 (1.8–5.5)	<0.001
**Age group**					
1 to < 5 years	24.9 (109/437)	Reference		-	-
< 1 year	21.7 (13/60)	1.2 (0.6–2.3)	0.64	-	-
**Nutritional Status**					
Well nourished	*	Reference		Reference	
Underweight	27.8 (20/72)	1.2 (0.7–2.1)	0.55	-	-
Severe underweight	10.5 (2/19)	0.4 (0.1–1.7)	0.21	-	-
Wasting	20.9 (9/43)	1.2 (0.6–2.6)	0.71	-	-
Severe Wasting	20.9 (9/43)	1.2 (0.5–2.5)	0.85	-	-
Stunting	24.1 (19/79)	1.2 (0.7–2.1)	0.57	0.5 (0.2–1.1)	0.09
Severe stunting	34.1 (28/82)	1.9 (1.1–3.3)	0.01	0.6 (0.2–1.8)	0.40
**Fever**					
Fever within 24 hours	50.0 (3/6)	3.1 (0.6–15.6)	0.16	-	-
History of fever in last month	57.1 (12/21)	4.4 (1.8–10.8)	0.001	3.6 (1.3–10.1)	0.02
**Malaria**					
Negative	18.7 (80/427)	Reference		Reference	
*P*. *falciparum*	78.9 (15/19)	16.3 (5.3–50.3)	<0.0001	10.0 (3.1–32.6)	<0.0001
*P*. *vivax*	52.2 (24/46)	4.7 (2.5–8.9)	<0.0001	3.6 (1.9–7.0)	<0.0001
Mixed Infections	66.7 (2/3)	8.7 (0.8–97.1)	0.079	-	-
**Helminthiasis**					
Negative	20.8 (32/154)	Reference		Reference	
Any worms	30.6 (30/98)	1.7 (0.9–2.9)	0.10	-	-
*Ascaris lumbricoides*	30.6 (22/72)	1.7 (0.9–3.1)	0.13	-	-
*Trichuris trichiura*	32.1 (17/53)	1.8 (0.9–3.6)	0.13	-	-
Hookworm	41.2 (14/34)	2.6 (1.2–5.8)	0.03	1.7 (0.8–3.8)	0.17
**Co-infections**					
Single Infections	29.8 (28/94)	Reference		-	-
Malaria and Helminth	63.2 (12/19)	4.0 (1.4–11.3)	0.008	1.53 (0.28–8.22)	0.620

Note:

*the proportion depends on nutritional status calculation method used

Of all the nutritional status indices, only severe stunting was associated with an increased risk of anaemia (OR = 1.9 [95%CI, 1.1–3.3] compared to well-nourished children, p = 0.012). The prevalence of anaemia was 34.1% (28/82) in children with severe stunting and 21.1% (65/308) in those with normal height according to age; p = 0.02.

Falciparum and vivax malaria were both significant risk factors for anaemia in children under five with ORs of 16.3 (95%CI, 5.3–50.3) and 4.7 (95%CI, 2.5–8.9) respectively, p<0.001; Table 5. Asymptomatic falciparum and vivax parasitaemia were associated with 4.3 times (95%CI, 2.0–9.4) and 2.9 times (95%CI, 1.7–4.9) greater risk of having anaemia respectively compared to children without parasitaemia (p<0.001). Anaemia was present in 41.2% (14/34) of children with hookworm infestation compared to 22.1% (48/217) of those without hookworm (OR of 2.6 [95%CI, 1.2–5.8], p = 0.026). Co-infections of *Plasmodium* and STHs occurred in 7.1% (19/269) of the children with available data. Compared to monoinfection the presence of both *Plasmodium* and STH was associated with a greater risk of anaemia (OR 4.0 [95%CI, 1.4–11.3], p = 0.008) and severe anaemia (OR 8.6 [95%CI, 1.3–55.8], p = 0.024).

## Discussion

In Papua, Indonesia, anaemia exerts a significant morbidity particularly in young children. Previous hospital based studies have highlighted the importance of undetected, untreated or inadequately treated malaria as a key contributor to reduction in haemoglobin concentrations, [3, 9]. The cross sectional study presented here provides additional insights into the importance of *Plasmodium* infection and the importance of soil transmitted helminths. *Plasmodium* infection was present in 12% of children under 5 years old, most of whom were asymptomatic, with *P*. *vivax* the dominant species of infection. Helminth carriage was common and when present with malaria doubled the risk of anaemia.

The aetiology of anaemia from malaria and helminth carriage is well described and is attributable to a combination of chronic blood loss, haemolysis and haemopoietic suppression. Associated nutritional deficiency is also likely to play a key role. Although in our study general measures of stunting and wasting were not significant risk factors, in the subset of individuals tested, microcytosis was present in almost 75%, suggesting a potential contribution of iron deficiency. Recent studies have demonstrated that increased hepcidin level and associated impaired intestinal iron absorption may play an important role in the development of anaemia in children with asymptomatic *Plasmodium* infection and may also play a role in *P*. *vivax* infections [30].

The high proportion of asymptomatic malaria in young children in this region suggests that clinical immunity develops at an early age in both *P*. *falciparum* and *P*. *vivax* infections [3, 31], although parasitaemias were low and it is plausible that the survey detected an early phase of infection prior to development of symptoms. The burden of *P*. *vivax* is particularly apparent in children under 5 years old, with the prevalence (8.8%) more than twice that of *P*. *falciparum* (3.9%). *Plasmodium vivax* peripheral parasitaemias were lower than in *P*. *falciparum* infections and children with *P*. *vivax* more likely to be asymptomatic. However the relapsing nature of *P*. *vivax* infections is an important determinant of anaemia in the first year of life [3] and is a major challenge for healthcare providers and clinicians since current National guidelines preclude the use of primaquine for infants aged less than 1 year [32]. In addition, the 2015 WHO guidelines only suggests primaquine use in in infants older than 6 months old [33].

Soil transmitted helminths (STHs) were present in almost half of the children by 5 years of age, and of these, almost half had multiple infestations. Stool sampling was undertaken in less than half of the children. The characteristics of those with and without stool samples (age, sex and parental socioeconomic status) was similar except for ethnicity, with 60% of Papuans were without stool sampling compared to 51% in non Papuan children. Papuan ethnicity was a major independent risk factor for helminthiasis raising the prevalence 2 to 4 fold, hence our analysis is likely to have underestimated the true burden of helminth infection.

The presence of hookworm was particularly important, increasing the risk of anaemia 2.6 fold. Once again the true burden is likely to be actually higher since Kato Katz has low sensitivity in diagnosing hookworm eggs [34]. Hookworm infestation is more prevalent in later childhood and early adulthood and is associated with chronic intestinal blood loss [14] [35]. Although less common in infants than older children, the prevalence of hookworm rose rapidly in the first year of life. Although our study did not quantify hookworm carriage in older children, the presence of this parasite at such an early age is interesting and may reflect earlier exposure to soil [35].

In Papua, children with malaria and STH co-infection were four times as likely to have anaemia compared to those having mono-infection with either pathogen, and at almost nine fold greater risk of severe anaemia. Although the frequency of co-infection was relatively low (7.1%), the presence of co-infection and its association with anaemia is likely to have a significant detrimental effect on the child’s health and development [14]. Similar findings were observed in Africa, where *P*. *falciparum* and hookworm comorbidity in preschool children was associated with lower haemoglobin concentrations compared to those having single infection [36]. On the other hand, school aged children with *P*. *falciparum* infections and hookworm infestation had lower risk of developing iron deficiency anaemia [37]. Although not generally recognized as a major cause of anaemia, in our study ascaris and trichuris as well as hookworm were associated with twice the risk of anaemia, although after controlling for ethnicity only hookworm remained a significant factor.

Dysregulation of cellular immune responses in STH infection [38–40] may down regulate protective immune responses to *Plasmodium* spp. in a complex way, increasing susceptibility to falciparum malaria without resulting in severe symptoms [36, 41, 42]. Although there are few studies from vivax-endemic areas, the interplay with *P*. *vivax* infection may be different. Heavy ascariasis infestation reduced the risk of symptomatic *P*. *vivax* malaria in one study of children aged 2–14 years old [43]. In another, STH infection protected against a further reduction in haemoglobin during acute vivax malaria [44]. Studies of immune responses, STH and malaria remain inconclusive and are not informative for *P*. *vivax* since most have been conducted in sub-Saharan Africa where *P*. *vivax* is not endemic [42, 45–49].

The sample size of our study was designed to evaluate the impact of antimalarial treatment policy by comparison with a prior survey conducted in 20015. The extensive data collected in the second survey enabled us to define the effect of asymptomatic malaria and STH infections to anaemia in children under-fives. The sample size of this study was sufficient to test the association of malaria, STH and anaemia [24]. However our study has a number of limitations. Firstly, the relative contribution of recurrent or chronic *Plasmodium* infection and STHs to the development of anaemia could not be examined in this cross sectional study. However in this population, individuals with a history of fever in the previous 30 days is a useful indicator of symptomatic malaria and in the cross sectional surveys was associated with a 3.6 fold higher risk of anaemia, suggesting a likely contribution of recurrent malaria. Secondly, iron status was not quantified so the contribution of iron deficiency could not be addressed. However in the subset of patients in whom data were available, three quarters of children with anaemia had microcytic erythrocytes suggesting iron deficiency and/or infections in the underlying aetiology.

In conclusion, asymptomatic *Plasmodium* infection and malaria/helminth coinfection are important risk factors for anaemia in young children. STHs appear to increase a child’s risk of *P*. *vivax* parasitaemia. Operational studies are needed to define the effectiveness of combined malaria and STH control programs to prevent their combined contribution to anaemia in early life and associated morbidity, mortality and cognitive impairment. Our study suggests that deworming has potential to reduce *P*. *vivax* infections. In the case of malaria, the risks and benefits of malaria screening or preventive treatment in infancy in areas endemic for *P*. *falciparum* and *P*. *vivax* warrants careful examination [50].

## Supporting Information

S1 Dataset Timika Household Survey. (XLSX)
